# Inflammatory biomarkers responses after acute whole body vibration in fibromyalgia

**DOI:** 10.1590/1414-431X20176775

**Published:** 2018-03-01

**Authors:** V.G.C. Ribeiro, V.A. Mendonça, A.L.C. Souza, S.F. Fonseca, A.C.R. Camargos, V.K.S. Lage, C.D.C. Neves, J.M. Santos, L.A.C. Teixeira, E.L.M. Vieira, A.L. Teixeira, B. Mezêncio, J.S.C. Fernandes, H.R. Leite, J.R. Poortmans, A.C.R. Lacerda

**Affiliations:** 1 Programa Multicêntrico de Pós-Graduação em Ciências Fisiológicas, Sociedade Brasileira de Fisiologia, Diamantina, MG, Brasil; 2 Programa de Pós-Graduação em Reabilitação e Desempenho Funcional, Universidade Federal dos Vales do Jequitinhonha e Mucuri, Diamantina, MG, Brasil; 3 Departamento de Fisioterapia, Faculdade de Ciências Biológicas e da Saúde, Universidade Federal dos Vales do Jequitinhonha e Mucuri, Diamantina, MG, Brasil; 4 Departamento de Fisioterapia, Escola de Educação Física, Fisioterapia e Terapia Ocupacional, Universidade Federal de Minas Gerais, Belo Horizonte, MG, Brasil; 5 Escola de Medicina, Universidade Federal de Minas Gerais, Belo Horizonte, MG, Brasil; 6 Escola de Educação Física e Esporte, Universidade de São Paulo, São Paulo, SP, Brasil; 7 Departamento de Agronomia, Faculdade de Ciências Agrárias, Universidade Federal dos Vales do Jequitinhonha e Mucuri, Diamantina, MG, Brasil; 8 Faculty of Motor Sciences, Université of Libre de Bruxelles, Brussels, Belgium

**Keywords:** Biomarkers, Fibromyalgia, Clinical trial, Inflammation, Acute exercise

## Abstract

The aims of this study were 1) to characterize the intensity of the vibration stimulation in women diagnosed with fibromyalgia (FM) compared to a control group of healthy women (HW) matched by age and anthropometric parameters, and 2) to investigate the effect of a single session of whole body vibration (WBV) on inflammatory responses. Levels of adipokines, soluble tumor necrosis factor receptors (sTNFr1, sTNFr2), and brain-derived neurotrophic factor (BDNF) were determined by enzyme-linked immunosorbent assay. Oxygen consumption (VO_2_) was estimated by a portable gas analysis system, heart rate (HR) was measured using a HR monitor, and perceived exertion (RPE) was evaluated using the Borg scale of perceived exertion. Acutely mild WBV increased VO_2_ and HR similarly in both groups. There was an interaction (disease *vs* vibration) in RPE (P=0.0078), showing a higher RPE in FM compared to HW at rest, which further increased in FM after acute WBV, whereas it remained unchanged in HW. In addition, there was an interaction (disease *vs* vibration) in plasma levels of adiponectin (P=0.0001), sTNFR1 (P=0.000001), sTNFR2 (P=0.0052), leptin (P=0.0007), resistin (P=0.0166), and BDNF (P=0.0179). In conclusion, a single acute session of mild and short WBV can improve the inflammatory status in patients with FM, reaching values close to those of matched HW at their basal status. The neuroendocrine mechanism seems to be an exercise-induced modulation towards greater adaptation to stress response in these patients.

## Introduction

Fibromyalgia (FM) is characterized by chronic widespread pain of more than 3 months duration (1). Clinical studies have observed that FM is associated with immune dysregulation of proinflammatory biomarkers, affecting the neural function of pain-related neurotransmitters (2). Blood interleukin (IL)-8 level is increased in FM (2) suggesting that IL-8 could be an “inflammatory marker” for FM syndrome (2). Tumor necrosis factor (TNF) is another potent cytokine, secreted mainly by macrophages, with mostly proinflammatory and catabolic actions, together with high levels in the serum of FM subjects (3). This marker has soluble receptors (sTNFR1 and sTNFR2) which compete with the surface receptor by binding to TNF and modulating its inflammatory activity, and it has been suggested that they function as physiological attenuators of the degradative activity of TNF (4). Whereas the soluble receptors are generally more stable in the circulation than the cytokines, the former can be more reliable markers of chronic inflammation (5).

Several factors termed adipokines, mainly produced in the adipose tissue have been found to be important regulators in both inflammation and nutrition, which may affect neuroendocrine regulation of pain and fatigue through several pathways (6). Adiponectin detects metabolic stress and modulates the metabolic adaptation targeting the innate immune system under pathological conditions, such as FM, demonstrating that this hormone seems to play an important role in regulating immune function during inflammation incidents (7).

Other important adipokines in metabolic and inflammatory processes are leptin and resistin. Leptin plays a regulatory role in immunity, inflammation, and hematopoiesis. Leptin and its receptor share structural and functional similarities with the interleukin-6 family of cytokines, and they affect cytokine production, activation of monocytes/macrophages, wound healing, angiogenesis, and hematopoiesis (8). The role of resistin in the metabolic profile is unclear; however, its role in inflammatory processes is stimulated by proinflammatory cytokines such as TNF and IL-6 (9). Moreover, they may induce the expression of these cytokines in adipose tissue and peripheral blood mononuclear cells (10). Therefore, it might be speculated that those adipokines may represent a link between inflammation and metabolic signaling.

Brain-derived neurotrophic factor (BDNF) is also intimately connected with central and peripheral molecular processes of energy metabolism and homeostasis (11). It is considered an essential neurotrophin synthesized by skeletal muscle cells in response to contraction stimulation together with enhancing fat oxidation via activation of adenosine monophosphate-activated protein kinase (12). Neurotrophin expression is usually enhanced in chronic inflammatory diseases because of its role in energy homeostasis (13). However, the current literature has evidenced that serum levels of BDNF did not differ significantly when comparing FM patients and controls (14).

Several therapies have been investigated to treat the symptoms associated with FM. To the best of our knowledge, only two studies have evaluated the acute effects of exercise in patients with FM. Bote and colleagues (15) demonstrated that mild cycling improve the inflammatory and stress status of FM. However, exhaustive exercise cannot be considered an anti-inflammatory stimulus (16).

Whole body vibration (WBV) has been proposed as a beneficial exercise modality for the treatment of FM (17,18) and other chronic inflammatory diseases (19). However, no study to date investigated whether this type of therapy could improve the inflammatory status in patients with FM. Although contraindications for exercise in patients with FM have not been described, it is mandatory to define the time and intensity of exercise programs to obtain responses that lead to anti-inflammatory effects (1).

The objectives of this study were: 1) To characterize the intensity of vibration stimulation in women diagnosed with FM as compared to a control group of healthy women (HW), matched by age and anthropometric parameters and 2) to investigate the effect of a single bout of WBV on inflammatory biomarker levels (adiponectin, sTNFr1, sTNFr2, resistin, leptin, BDNF, and IL-8).

## Material and Methods

### Ethical statement

This is a prospective 1:1 case-control paired study that assessed variables before and immediately after an acute session of WBV. This study was conducted in accordance with the ethical principles for research involving humans (principles of the Declaration of Helsinki) and received approval from the Ethics Committee of the Universidade Federal dos Vales do Jequitinhonha e Mucuri (No. 1.461.311), and Brazilian Guidelines (Res. CNS 196/96, No. RBR-36d8nf). All participants were informed about the study procedures and provided their written consent to participate in this study.

### Study design and subjects

The volunteers were recruited from April to June, 2016 through the waiting list of physiotherapy from Universidade Federal dos Vales do Jequitinhonha e Mucuri, Basic Health Units, Specialized Center for Rehabilitation, and announcements on the radio of Diamantina, MG, Brazil.

**Figure 1.**
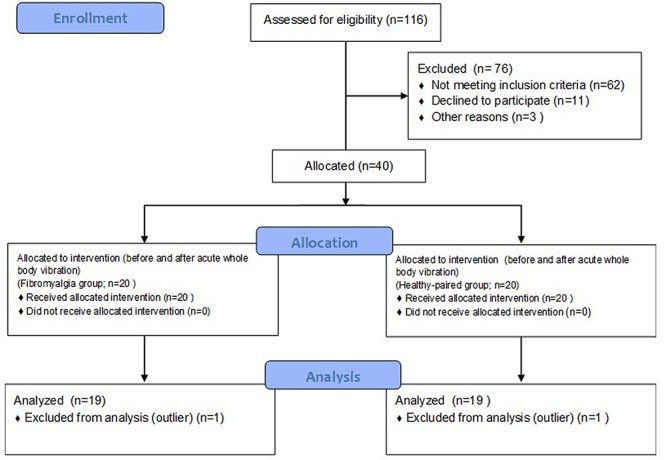
Study flowchart.

A sample size of 38 volunteers was required to meet the significance level of 0.05 and power of 80% before the beginning of this study. Thus, a total of 116 women were initially assessed for eligibility. Of these, 76 were excluded as they did not meet the inclusion criteria (n=62), declined to participate (n=11), or for other reasons (n=3). The remaining 40 women were allocated to a fibromyalgia group (n=20) or a healthy-paired group (n=20). Both groups were submitted to vibration exposure. However, one subject from each group was excluded because blood biomarkers were identified as outliers through Grubbs' test, also called the ESD method (Figure 1). Thus, nineteen women diagnosed with FM according to the American College of Rheumatology criteria (1) were enrolled in the study. Some anthropometric, demographic, and clinical data are shown in Table 1. Participants were asked to fill out a questionnaire about their lifestyle (diet, habits, etc.), medication, and other previous or current concomitant illnesses. The subjects completed the “Physical Activity Readiness Questionnaire (PAR-Q)” (15) as well as the “Fibromyalgia Impact Questionnaire” (FIQ) (20). They were also characterized by the “Beck Depression Inventory and the sitting-rising test” (21,22) in order to confirm that all FM volunteers had a chronic and stable disease status. The exclusion criteria were the presence of any concomitant disease that could be exacerbated by physical activity, pregnancy, inflammatory diseases, and degenerative, joint, respiratory, or cardiovascular disease. Other exclusion criteria were: 1) volunteers being followed by a psychiatrist or those who performed physical activity more than two times per week; 2) subjects who displayed any of the possible contraindications for WBV stimulus, such as acute hernia, stroke, diabetes, epilepsy, metabolic or neuromuscular diseases, orthopedic and prosthetic lesions (23); and 3) women who were taking oral or topical immunosuppressive medication (corticosteroids) or anti-cytokine therapy that could influence the level of cytokines. The control group was composed of 19 healthy women matched by age, body mass, height, and medication intake, without any pain disorders or infectious illness. The same requirements were applied to both groups. All volunteers were physically inactive, did not participate in exercise programs during the previous 24 months, and were non-smokers and non-consumers of alcohol.

**Table 1.** Characteristics of study subjects.

	HW (n=19)	FM (n=19)	P
Age (years)	51.05±1.90	52.16±1.81	0.61
Weight (kg)	73.38±3.09	74.79±3.20	0.76
Height (m)	1.58±0.02	1.58±0.01	0.68
BMI (kg/cm^2^)	29.65±1.28	29.71±1.09	0.97
Tender points	0.85±0.45	14.1±0.72*	<0.0001
BDI	10.90±1.16	22.10±1.88*	0.0002
SUT	12.84±0.38	8.57±0.79*	0.0002
FIQ	-	75.58±4.40	-
Time from diagnosis (years)	-	>2**	-
IL-8 (pg/mL)	4.13±0.74	7.42±1.62	0.006

HW: healthy women; FM: fibromyalgia; BMI: body mass index; BDI: Beck Depression Inventory; FIQ: Fibromyalgia Impact Questionnaire; SUT: Sit-up test; IL8: interleukin 8. **100% of volunteers. Data are reported as means±SE. *P<0.05 (Tukey’s test).

### Procedures

One week before the trial, all the volunteers were familiarized with the vibrating platform. The session was scheduled during the day and carried out in a reserved room with the temperature maintained at approximately 23°C. Anthropometric characterization, evaluation of tender points, questionnaires, and tests to evaluate the muscular performance of the lower limbs (sitting and standing) were also carried out. The evaluation was conducted by the same investigator to ensure equal instruction. Analysis of the questionnaires was performed by a blind researcher (24).

Measurement of body weight was performed by an anthropometric mechanical scale, equipped with a stadiometer (Seca, Germany). The body mass index (BMI) was determined as the total body weight divided by height squared (kg/m^2^) (25).

The assessment of tender points was performed by palpation. A tender point was considered positive when painful discomfort was triggered after digit pressure of around 4 kg/cm (1).

The health status, functional capacity, and main symptoms of FM were assessed using the “Brazilian version of the FIQ” (20). Evaluation of depression was performed using the Beck Depression Inventory (21). Muscle strength of the lower limbs was evaluated by the sit-and-stand chair test (22).

### Trial

All volunteers arrived at the experimental center at 7:30 am, after fasting for at least 8 h, including not taking regular medication (15). The experimental protocol began with the subjects remaining at rest for 30 min. During this period, the volunteers were instructed to remain seated, and not to perform sudden movements.

The vibration exposure consisted of performing squatting exercises with a vibration stimulus (frequency of 40 Hz and amplitude of 4 mm) performed on a commercial model of vibration platform (FitVibe, GymnaUniphy NV, Belgium). This vibration frequency and amplitude was selected because this prototype renders an acceleration range of 2–5 *g* (26). Acceleration is a variable used to quantify the intensity of the vibration stimulus (19).

During vibration stimulus, the volunteers performed 8 series of 40 s of squatting exercises. During each exercise series, the volunteer was instructed, by the same examiner blind to the group assignments of the participants, to perform a semi complete extension (ca. 10°) to a 60° angle knee bend. The 60° angle was measured for each volunteer by using a universal goniometer before initiating the exercise series, and a barrier was placed at the gluteal region to limit the flexion degree of the knees. To control the time of each squat, an examiner instructed the individuals to bend their knees to an 60° angle for 3 s and then to an 10° angle for 3 s, over the 40 s of each series, for a total of 5 repetitions each. The participants were also instructed to remain in the correct position with their feet on the platform and their spine, arms, and head also in their instructed position (23).

### Inflammatory biomarkers

Peripheral blood samples were collected before and immediately after exercise for each volunteer, and plasma was prepared and stored at −80°C until required. IL-8 level was measured using the cytometric bead arrays kit (BD Bioscience, USA) according to the manufacturer's protocol. Samples were acquired in a FACSCanto flow cytometer (BD Biosciences) and analyzed using the FCAP Array v1.0.1 software (Soft Flow Inc., Hungary). The detection limits were 0.2 pg/mL for IL-8 level.

Adiponectin, plasma soluble TNF receptor (sTNFR1, sTNFR2), leptin, resistin, and BDNF levels were measured using conventional sandwich enzyme-linked immunosorbent assay kits (DuoSet, R&D Systems, USA), according to the manufacturer's instructions. The detection limits were 5.0 pg/mL for all kits.

### Evaluation of oxygen consumption and heart rate

To measure oxygen consumption, a K4b2 portable gas analysis system (Cosmed, Italy) was used to transmit breath-by-breath data to a computer throughout the experimental protocol. HR was measured and registered after 30 minutes at rest and immediately after vibration exposure (Polar RS800sd) (23).

The Borg Scale of Perceived Exertion (RPE) and heart rate were measured and registered after 30 minutes at rest and immediately after vibration exposure (27).

### Statistical analyses

Data are reported as means±SE. These are two experiments with common treatments. Tukey’s test was used to compare the mean with a 5% significance level.

The model used (28) was: y_ijk_=m + a_i_ + b_j_ + ab_ij_ + r_k(j)_ + e_ik(j)_ where: m=general mean; a_i_=effect of vibration (before and after) i; b_j=_effect of the illness (fibromyalgia and control groups) j; ab_ij_=effect of the interaction between these two factors; r_k(j)=_effect on patients (repetitions) within groups (control and fibromyalgia); e_ik(j)=_weighted average error.

## Results

There were no differences between the groups FM and HW with regard to age, body mass, height, or BMI (P>0.05). The FM group had significantly greater tender points than the HW group (P<0.0001). The FM group had higher scores on BDI (49%), where scores of 17–27 are characterized as mild depression. The minimum time from diagnosis in the FM group was two years. A score of 75.58±4.40 was found in the FM group for the FIQ. Considering the sit-up test, the HW group showed a higher number of repetitions (12.84±0.38) compared to the FM group (8.57±0.79), characterizing reduced muscle performance of lower limbs in the FM group. Two volunteers of the FM group failed to make any repetition in this test (Table 1).

**Figure 2.**
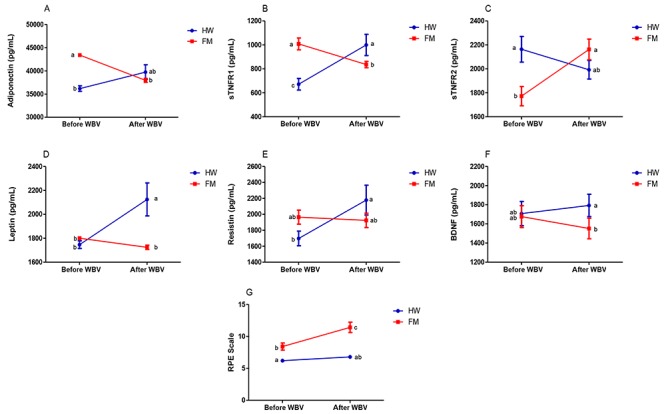
Interaction between biomarkers (*A*-*F*) before and after whole body vibration (WBV). Blue represents the healthy women group (HW, n=19) and red the fibromyalgia women group (FM, n=19). Adiponectin (*A*), plasma soluble tumor necrosis factor-receptors 1 (sTNFR1) (*B*) and 2 (sTNFR2) (*C*), leptin (*D*), resistin (*E*), brain-derived neurotrophic factor (BDNF) (*F*), and Borg Scale of Perceived Exertion (RPE, *G*) in healthy women and in fibromyalgia women (FM; n=19). Data are reported as means±SE. P<0.05, different small letters indicate significant differences (Tukey’s test).

Mild vibration exposure increased oxygen consumption and heart rate similarly in both groups. The FM group had a higher RPE, which further increased after vibration stimulus, than the HW group at rest, which remained unchanged (Table 2). However, there was an interaction in the Borg scale of perceived exertion (p=0.0078), shown in (Figure 2G).

**Table 2.** Oxygen consumption (VO_2_) and heart rate (HR) in HW (n=19) and FM (n=19) groups before and during stimulus of WBV.

	VO_2_ (mLO_2_·kg^-1^·min^-1^)	HR (bpm)	RPE scale
WH	2.69±0.17	73±1.65	6.21±0.12
WH+WBV	6.33±0.44^+^	93±2.37^+^	6.79±0.27
FM	3.10±0.13	80±2.38	8.42±0.54*
FM+WBV	7.31±0.28^+^	97±2.64^+^	11.42±0.80^+^**

WH: healthy women; FM: fibromyalgia; WH + WBV: healthy women during whole body vibration; FM + WBV: fibromyalgia women during whole body vibration; HR: heart rate; bpm: beats per minute; RPE: Borg Scale of Perceived Exertion. Data are reported as means±SE. *P<0.05 FM *vs* WH. ^+^P<0.05 WBV effect. **P<0.05 FM+WBV *vs* WH+WBV (Tukey’s test).

**Figure 3.**
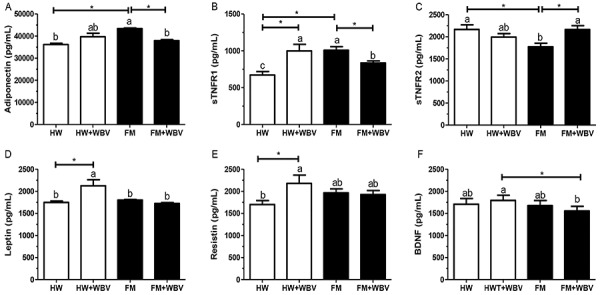
Effect of acute session of whole body vibration (WBV) on plasma levels of adiponectin (*A*), soluble tumor necrosis factor- receptors 1 (sTNFR1) (*B*) and 2 (sTNFR2) (*C*), leptin (*D*), resistin (*E*), and brain-derived neurotrophic factor (BDNF) (*F)* in healthy women (HW; n=19) and in fibromyalgia women (FM; n=19). Data are reported as means±SE. P<0.05, different small letters and asterisks indicate significant differences (Tukey’s test).

Subjects with FM showed higher plasma levels of IL-8 at rest regardless of associated depression (4), demonstrating the occurrence of chronic inflammatory disease (FM: 7.42±1.62 pg/mL; HW: 4.13±0.74 pg/mL; P=0.006). Moreover, the FM group presented increased plasma levels of adiponectin (FM: 43385.80±274.98 pg/mL; HW: 36180.61±594.53 pg/mL; P=0.010), and sTNFR1 (FM: 1008.28±49.09 pg/mL; HW: 671.51±48.24 pg/mL; P=0.048), and decreased plasma concentrations of sTNFR2 (FM: 1772.02±80.39 pg/mL; HW: 2163.35±106.71 pg/mL; P=0.005) compared to the HW group. There was no difference between the groups for plasma levels of leptin, resistin, or BDNF (P>0.05) (Figure 3).

The vibration exposure decreased plasma levels of adiponectin and sTNFR1 and increased levels of sTNFR2 in the FM group. Furthermore, it increased leptin, resistin, and sTNFR1 plasma levels in the WH group (Figure 3). There was an interaction in plasma levels of adiponectin (P=0.0001), sTNFR1 (P=0.000001), sTNFR2 (P=0.0052), leptin (P=0.0007), resistin (P=0.0166), and BDNF (P=0.0179; Figure 2A-F).

## Discussion

We highlighted the effects of a single acute session of WBV on improving the inflammatory status in patients with FM, redirecting parameters towards metabolic homeostasis, and reaching values close to those of anthropometric parameter and age-matched HW at their basal status. To date, this is the first study that investigated the effects of an acute WBV stimulus on the inflammatory response in women with FM.

Although vibration exposure increased oxygen consumption and heart rate equally in both groups, the intensity used was low according to the recommendations of the American College of Sports Medicine (ACSM) and American Heart Association (AHA) (29), representing an additional increase of only 1 MET (3 mL O_2_·kg^-1^·min^-1^). This result is consistent with the previous findings of our team that showed an increase of 2 METS, corresponding to a light walk, in a study that evaluated the effect of acute vibration exposure in the elderly (23). Furthermore, although the control group was composed of healthy women, they were about 51 years old, sedentary and overweight.

The FM group exhibited greater perceived exertion at rest and a further increase after acute WBV, whereas it remained unchanged in the HW. However, the exercise was characterized as mild for both groups. Moreover, individuals with FM had lower physical performance in the sit-up test, and attenuated physical and emotional scores in the FIQ, supporting the fact that they have a degenerated exercise perception.

Recent hypotheses of FM etiologies have highlighted the inflammatory disorders accompanied by changes in the neuro-immunoendocrine system (15). Studies have shown that individuals with FM have increased IL-8 plasma levels compared to healthy subjects (2,15). In this study, women with FM showed higher plasma levels of IL-8 and sTNFR1 at baseline. Thus, it is possible that the individuals in FM group have an “inflammatory status” inherent to the disease (3).

Moreover, the FM group showed higher plasma levels of adiponectin at rest. Initially this result seems paradoxical, since most studies indicate the anti-inflammatory effect of this adipokine (30). The effect of adiponectin on blood circulation depends on a complex interaction between the disease state and the nature of the inflammatory stimulus (31). However, whether adiponectin acts as an anti or pro-inflammatory factor is still a matter of debate (32). Adiponectin detects metabolic stress and modulates the metabolic adaptation targeting the innate immune system under pathological conditions, such as FM, demonstrating that this hormone appears to play an important role in regulating immune function in inflammation status (7). Studies have shown that increases in adiponectin levels are associated with worsening physical performance via interaction with adipoR1 and adipoR2 receptors (31). The adipoR1 receptor is abundantly expressed in vascular endothelial cells and skeletal muscle cells, where it regulates glucose and lipid metabolism, while adipoR2 receptor, which acts as a moderate affinity receptor, is mainly expressed in the liver (31). Simultaneous disruption of both receptors nullifies adiponectin binding, resulting in glucose intolerance and insulin resistance (33). Thus, the higher levels of adiponectin in the FM group could contribute to energy imbalance, with structural and functional changes in muscle fibers and hormonal imbalances resulting in a state of myopathy.

The plasma adiponectin levels did not change immediately after acute WBV stimulus in asymptomatic subjects. These results are consistent with current literature (34). Women with FM, who showed higher plasma level of adiponectin at rest, had a reduction in adiponectin levels after WBV stimulus. Although mild and of short duration, this stimulus appears to be beneficial, since it indicates a reduction of adiponectin receptor resistance in the peripheral muscle, contributing to metabolic homeostasis.

The FM group also showed higher plasma levels of sTNFR1 at rest. It appeared that this increase occurred as an attempt to control the chronic systemic inflammation. Thus, we hypothesized that the increase in plasma levels of sTNFR1 occurred while trying to promote inhibition of catabolic action of TNF, inactivating the consequent inhibition of its binding to membrane receptors, together with a reduced supply of its own membrane receptors. sTNFR1 soluble receptor is derived by the proteolytic cleavage of plasma membrane-expressed sTNFR1 catalyzed by TNF converting enzyme (TACE), modulating its inflammatory activity probably by attenuating the harmful activity of TNF (4). However, since adiponectin and TNF inhibit each other (35), the increase in soluble receptor levels of sTNFR1 suggests that this control did not actually occur efficiently.

In healthy women, vibration exposure promoted an increase in plasma sTNFR1 levels. By contrast, the anti-inflammatory effects induced by mild stimulus in patients with FM could also be considered a positive effect on homeostatic adjustment involved in inflammation-stress feedback mechanisms (15).

Regarding sTNFr2 soluble receptor, we observed lower blood levels in women with FM at rest. It seems that hyper-adiponectinemia plays a role in reducing the secretion of this receptor (36). However, this mechanism remains unclear. Moreover, once the intracellular actions of TNF are mostly mediated by sTNFR1 and sTNFR2 (4), it is possible to infer that this marker did not act efficiently to maximize the association between TNF and sTNFR1.

Other important adipokines in the inflammatory response and potential regulators of metabolic function are leptin and resistin (37). The inflammatory and metabolic modulator role of adipokines may have influenced the similarity in blood baseline levels in subjects with FM and HW. However, in situations of metabolic imbalance and consequent alertness, such as during acute stimulation WBV, blood levels of this biomarker increase systemically (38). Since patients with FM have a constant metabolic and inflammatory stress, the turnover of leptin appears to have a higher threshold to stressful stimuli, which could explain the maintenance of blood levels of this biomarker after WBV stimulus in these patients (15).

Basal blood levels of resistin were similar between groups. This finding is similar with Bjersing and colleagues (6) study, which also identified stable baseline levels of these adipokines in overweight/obese subjects with FM. However, acute vibration exposure in healthy subjects induced an increase in the levels of these adipokines. Thus, in healthy matched-group, the activation of innate and/or inflammatory responses (considered a “state of alert”), which is indispensable to prevent and defend against pathogenic attacks, could be interpreted as a positive physiological adaptation against a situation of vulnerability to the body (15).

In addition to adipokines, BDNF levels did not differ between groups. This finding is in line with the current literature that has shown that serum levels of BDNF did not differ significantly between patients with FM and controls (14). The action of this neurotrophin is closely linked to central and peripheral molecular processes of energy metabolism together with homeostasis associated with induction of a cascade of neurotrophic and neuroprotective effects (11). However, its role in FM as a possible enhancer or attenuator of pain is still controversial. Thus, despite the fact that activation of NMDAR (N-methyl-D-aspartate: an inotropic receptor activated by glutamate/aspartate) by endogenous BDNF appears to contribute to hyperalgesia by increasing awareness of dorsal neurons, the current literature indicates the role of BDNF as a mediator of pain through downregulation of TrkB receptors involved in pain modulation. However, this mechanism is not well understood in patients with FM and other chronic diseases (39).

Although BDNF levels did not differ between groups in this study, acute vibration exposure reduced plasma levels of BDNF in FM group. The literature shows that BDNF is produced by skeletal muscle cells in response to contraction, enhancing fat oxidation via activation of AMP-activated protein kinase (12). Physical activity seems to be a key intervention to trigger the processes through which neurotrophins mediate energy metabolism and their neural plasticity. According to Knaepen and colleagues (11), of all neurotrophins, BDNF seems to be the most susceptible to exercise and physical activities. Considering that the turnover of BDNF after low-intensity stimulation in patients with FM was not assessed, an explanation of this finding is beyond the scope of this study. However, we propose that the action of this neurotrophin modulates towards a greater adaptation to stress response in these patients.

There are limitations in the interpretation of the results of our study. Despite the instruction to our volunteers in both groups to abstain from medication 8 h prior to the trial, it is not possible to completely exclude the effect of drugs on plasma levels of biomarkers. Moreover, we matched the group volunteers regarding the use of medications, especially the intake of glucocorticoids. Once there are many potential confounding factors in the level of cytokines (e.g., circadian rhythm, medication, physical activity, infections, body mass index, and mood state), we carefully and rigorously controlled the matching of FM and healthy women by age and anthropometric parameters. Finally, although some subjects in the FM group were taking antidepressant drugs that could activate monocytes and macrophages, increasing the production of anti-inflammatory cytokines (40), FM group showed a chronic inflammatory profile.

In conclusion, this study demonstrated that a single acute session of mild and short WBV can improve the inflammatory status in patients with FM, reaching values close to healthy anthropometrically- and age-matched HW at their basal status. The neuroendocrine mechanism seems to be an exercise-induced modulation towards greater adaptation to stress response in these patients.
